# Supplementary material for: Regulatory Effect of Bacillus subtilis on Cytokines of Dendritic Cells in Grass Carp (Ctenopharyngodon Idella)
Source: Int J Mol Sci. 2019 Jan 17;20(2):389. doi: 10.3390/ijms20020389 (PMC6359277; doi:10.3390/ijms20020389)
Supplement: Supplementary file 1 [file ijms-20-00389-s001.pdf]

Supplementary Figures

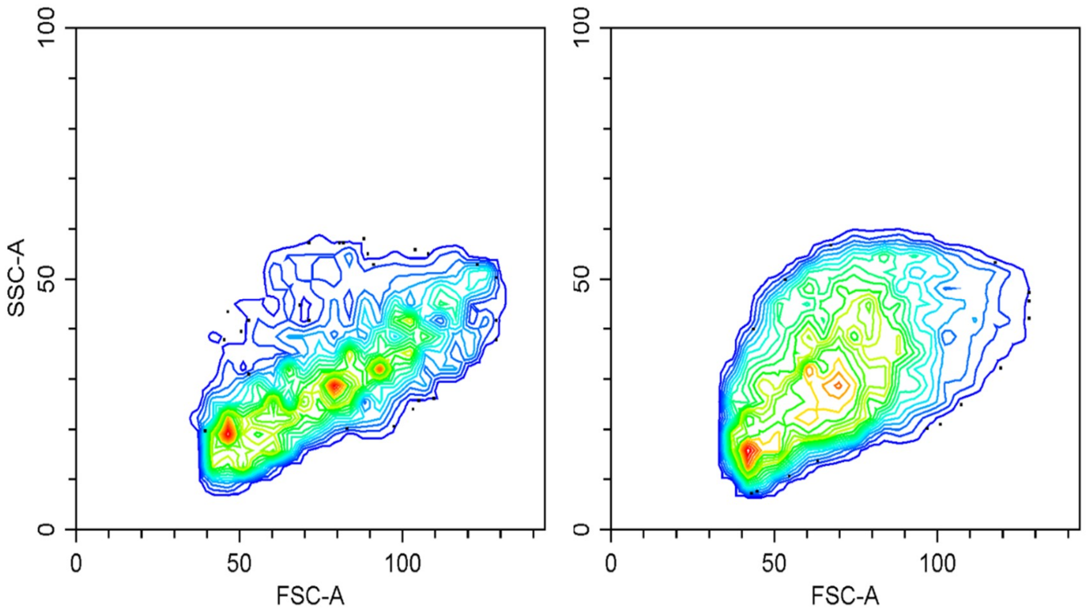

Supplementary Figure S1 | Flow cytometry forward/side scatter profile of cultured cells before (left pannel) and after enrichment (right pannel). Data are representative of three independent experiments. Typical purity after enrichment was more than 80%.

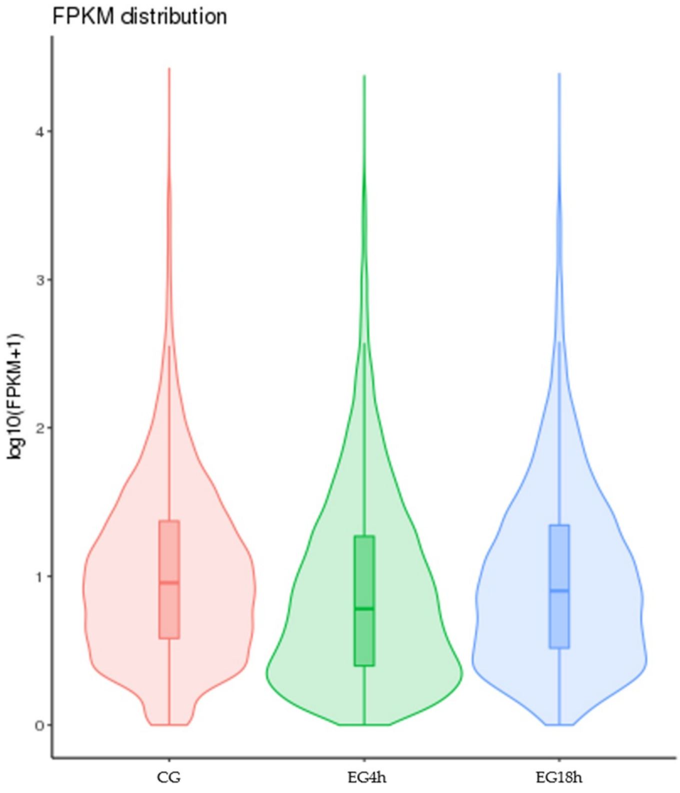

Supplementary Figure S2 | FPKM density distribution. The log10(FPKM+1) is used for the Y axis and the sample name is used for the X axis. Volin plots show the lowest and highest values in the distribution, the median, and the lower quartile and the upper quartile of a given set of data. Each group contains three independent samples.

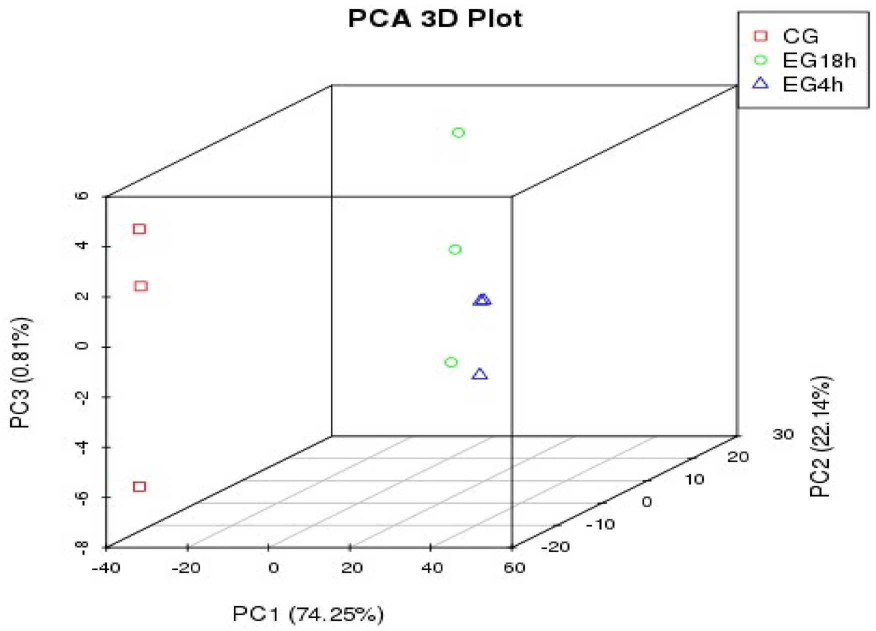

Supplementary Figure S3 | Principal Component Analysis (PCA) of the transcriptome expressed in CG, EG4h and EG18h. PCA was carried on all genes under investigation to determine the expression trends within the data set.
